# Does Intra-Operative IV Dexamethasone Reduce Postoperative Pain More Effectively With Nonopioids or Opioids?

**DOI:** 10.1016/j.joms.2025.09.022

**Published:** 2025-10-10

**Authors:** Ryan Look, Vincent B. Ziccardi, Tracy Andrews, Patricia Greenberg, Janine Fredericks-Younger, OARS Study Group

**Affiliations:** * Resident, Department of Oral and Maxillofacial Surgery, Rutgers School of Dental Medicine, Newark, NJ.; † Professor, Chair, and Associate Dean for Hospital Affairs, Department of Oral and Maxillofacial Surgery, Rutgers School of Dental Medicine, Newark, NJ.; ‡ Senior Biostatistician, Department of Biostatistics and Epideiology, School of Public Health, Rutgers University, Newark, NJ.; § Associate Professor and Associate Dean for Graduate Programs, Department of Dentistry, Rutgers School of Dental Medicine, Newark, NJ.

## Abstract

**Background:**

Corticosteroids reduce postoperative swelling and trismus. However, their role in pain management, particularly when combined with opioid versus nonopioid analgesics, is less understood.

**Purpose:**

The purpose of the study was to determine whether intra-operative corticosteroids reduce postoperative pain following third molar surgery and compare its effect on pain relief within nonopioid (NSAIDs and acetaminophen) and opioid analgesic treatment arms.

**Study Design, Setting, and Sample:**

This retrospective cohort study is based on the multicenter Opioid Analgesic Reduction Study (OARS) (n = 1,815). Participants who received oral corticosteroids (n = 41) were excluded.

**Predictor Variable:**

The primary predictor was the therapeutic treatment (opioid, nonopioid). Within each treatment arm, participants were grouped based on intra-operative steroid use (steroid, no steroid).

**Main Outcome Variable(s):**

Average pain (numeric rating scale: 0 to 10) was measured on postoperative days 1, 2, and 3 and entire postoperative period. Secondary outcomes included satisfaction with pain medication, unused analgesic tablets, use of rescue opioids, and emergency clinic visits.

**Covariates:**

Covariates included age, sex, race/ethnicity, preoperative pain and swelling, number of teeth extracted, impaction level, anesthesia type, use of antibiotics, and the administration of 0.5% bupivacaine with 1:200,000 epinephrine.

**Analyses:**

Mixed-effects models estimated the interaction of analgesics by steroid use by time with random effects for study sites and participants. Differences were assessed using *P* < .00625 to account for multiple comparisons.

**Results:**

The sample included 1,774 subjects with a mean age (SD) of 25.7(6.2) and 893(50.3%) were female. Analgesics by steroid groups were distributed as follows: nonopioid + no-steroid (626(35.3%)), opioid + no-steroid (635(35.8%)), nonopioid + steroid (265(14.9%)), and opioid + steroid (248(14.0%)). Steroid exposure was associated with lower day 1 postoperative pain in both the nonopioid group (mean difference 0.78; 99.375% CI 0.25 to 1.30; *P* < .001) and the opioid group (1.22; 0.68 to 1.77; *P* < .001). Across all days, the nonopioid + steroid group had the lowest pain scores, with a mean difference of 0.55 (−0.04 to 1.14; *P* = .026) compared with the nonopioid+ no steroid group.

**Conclusion:**

Intra-operative IV dexamethasone was associated with reduced pain for the first 24 hours following third molar surgery. Adding dexamethasone to either analgesic group improved pain control. Providers should consider incorporating intra-operative IV dexamethasone with postoperative nonsteroidal anti-inflammatory drugs and acetaminophen.

Effective postoperative pain management is a critical aspect of third molar surgery, one of the most common in-office procedures provided by oral and maxillofacial surgeons. ^1–4^ Often, third molars that necessitate removal are either impacted or infected, increasing the risk of postoperative complications. These complications often include, but are not limited to, pain, swelling, trismus, infection, alveolar osteitis, and nerve injury, specifically to the lingual and inferior alveolar nerves. Pain, swelling, and trismus are the result of the inherent inflammatory processes that arise from any surgical intervention. ^1–3^ To mitigate these inflammatory sequelae, a combination of pharmacological strategies is often employed comprising of corticosteroids, nonsteroidal anti-inflammatory drugs, acetaminophen, and opioids. ^5,6^ For the purpose of this paper, the terms steroids, corticosteroids, and dexamethasone will be used interchangeably.

Corticosteroids contain both glucocorticoid and mineralocorticoid sub-groups. Among their diverse effects, the anti-inflammatory actions of glucocorticoids are particularly relevant to surgical recovery. Through the inhibition of phospholipase A2, corticosteroids reduce inflammation by preventing the conversion of phospholipids to arachidonic acid, thereby preventing the formation of pro-inflammatory leukotrienes, cytokines, and prostaglandins. ^7–9^ Additionally, corticosteroids can stabilize cell membranes, prevent the release of inflammatory enzymes, and reduce capillary permeability.^8^ Collectively, these various mechanisms lead to the decrease in postoperative swelling and trismus. Of particular interest is the potential analgesic benefit of corticosteroids, mediated through its indirect inhibition of cyclooxygenase and its byproducts. ^3,10,11^

When managing postoperative pain, oral and maxillofacial surgeons most commonly prescribe nonsteroidal anti-inflammatory drugs (NSAIDs), acetaminophen, and opioids. ^12^ Like corticosteroids, NSAIDs target the arachidonic acid pathway. However, unlike corticosteroids, NSAIDs directly inhibit cyclooxygenase resulting in its excellent pain-reducing properties. ^13,14^ In comparison, acetaminophen, a very weak inhibitor of cyclooxygenase, exerts its analgesic properties through its metabolite N-acylphenolamine and its binding to TRPV1 receptors in both the brain and spinal column. ^14,15^ More commonly today, NSAIDs and acetaminophen are prescribed in combination as studies have shown this regimen to provide highly effective analgesia and be comparable, or superior, to opioids. ^14,16^ In contrast, opioids do not act on the cyclooxygenase pathway. Instead, it acts centrally through binding of opioid receptors and inhibiting the releasing of neurotransmitters.^17, 18^

Despite substantial evidence supporting the role of corticosteroids in reducing postoperative swelling and trismus, the extent to which their use impacts pain when combined with nonopioids (NSAIDs and acetaminophen) or opioids remains less clear. Given the shift toward minimizing opioid use in pain management, the purpose of this study was to determine whether intra-operative corticosteroids like dexamethasone reduce postoperative pain following third molar surgery. Additionally, it compared pain relief outcomes when corticosteroids were paired with nonopioid analgesics (NSAIDs and acetaminophen) versus opioid analgesics. We hypothesized that corticosteroids aids in reducing postoperative pain, and its combination with nonopioids would provide pain relief equal to, if not superior to, its combination with opioids.

The specific aims are three-fold: first, to evaluate the effect of intra-operative IV dexamethasone on postoperative pain after third molar surgery; second, to compare pain relief outcomes between subjects receiving nonopioid analgesics and those who received opioid analgesics, both with and without the addition of intra-operative steroids; and third, to assess secondary outcomes related to adverse events. By clarifying the role of corticosteroids in pain management, this study seeks to refine intra- and postoperative protocols, enhancing patient outcomes while reducing dependency on opioids.

## Methods

### STUDY DESIGN/SAMPLE

This is a retrospective cohort study utilizing data from the double-blind, prospective, sex-stratified, randomized quasi-pragmatic Opioid Analgesic Reduction Study (OARS) clinical trial, which was designed to evaluate the effectiveness of a combination of over-the-counter nonopioid analgesics (ibuprofen 400 mg and acetaminophen 500 mg) versus opioids (hydrocodone 5 mg/acetaminophen 300 mg) in managing postoperative pain following impacted third molar extraction.^19^ The OARS trial was approved by the institutional review board at Rutgers (protocol # 2020002299).

The OARS trial was conducted at 5 centers across the United States, located at Rutgers School of Dental Medicine, Newark, NJ; University of Illinois Chicago College of Dentistry, Chicago, IL; University of Maryland School of Dentistry, Baltimore, MD; University of Rochester School of Medicine and Dentistry, Rochester, NY; and University of Michigan School of Dentistry, Ann Arbor, MI. All participating centers followed standardized protocols to ensure consistency in data collection and patient care.

Prior to surgery, all participants were randomized into either the opioid or nonopioid (NSAIDs and acetaminophen) group. On study day 1, surgery was performed, and the need for intra-operative IV dexamethasone administration was determined by the treating providers. Postoperatively, participants were instructed to complete a twice daily electronic diary (morning and evening) to track their pain, amount of pain medication taken, and side effects experienced until their postoperative appointment, which was scheduled to occur between study day 5 and 15.

For this secondary retrospective cohort study, the original inclusion and exclusion criteria from the OARS trial were applied (see Table 1). However, 41 participants who received oral corticosteroids were excluded, as there were a variety of oral medications provided. All remaining participants either did or did not receive intra-operative IV dexamethasone.

### VARIABLES

The primary predictor was the therapeutic treatment (opioid, nonopioid). Within each treatment arm, participants were grouped based on intra-operative steroid use (steroid, no-steroid) as follows: nonopioid + no-steroid, nonopioid + steroid, opioid + no-steroid, and opioid + steroid.

The primary outcome was average postoperative pain on the first day and evening, second day and evening, and third day and evening following surgery when pain is most likely to be at its peak, as well as over the entire postoperative follow-up period. Pain was reported twice daily in electronic diaries using a numeric rating scale (NRS) from 0 to 10 (0 = no pain, 10 = worst pain imaginable). Secondary outcomes measured at the postoperative visit included satisfaction with pain medication (range from 1 = very satisfied to 5 = very dissatisfied), number of opioid tablets returned (range 0 to 20), need for emergent clinical visit (yes, no), and need for rescue medication (yes, no). Whether or not side effects (SEs) were experienced was collected using the daily electronic diary and dichotomized to represent whether participants ever report side effects over the postoperative period (yes, no).

Covariates included baseline demographics, preoperativ e pain measures, and surgical parameters. Baseline demographics included age (range 18 to 63), sex (female, male), race/ethnicity (non-Hispanic Black, non-Hispanic White, non-Hispanic Asian, Hispanic, and non-Hispanic other), study site, education level (some high School, high school graduation, associate’s degree, bachelor’s degree, master’s degree, and doctoral degree), and smoking history (do not smoke, less than a pack a day, a pack a day, more than a pack a day). Pre-operative pain measures included self-reported pre-operative swelling (none, mild, moderate, and severe), worst pain in the prior 24 hours (NRS scale, range 0 to 10), average pain in the prior 24 hours (NRS scale, range 0 to 10), and pain tolerance (NRS scale, range 0 to 10). Surgical parameters included minutes in surgery (range 3 to 150), total teeth extracted (range 1 to 4), maximum impaction level (partial or full-bony), anesthesia type (local only, oral/nitrous/conscious sedation, and general), use of intra-operative antibiotics (yes, no), use of intra-operative 0.5% bupivacaine with 1:200,000 epinephrine (Marcaine) (yes, no), and the most difficult surgical technique used during the procedure (forceps or elevator only, osteotomy only, osteotomy, and tooth sectioning).

### DATA COLLECTION METHODS

At the screening appointment (visit 0), patients were assessed to determine if they had at least one impacted mandibular third molar treatment planned for extraction. During this visit, informed consent was obtained, eligibility was evaluated based on inclusion and exclusion criteria, and participants provided self-reported medical histories.

On the day of surgery (visit 1), eligibility was reconfirmed through the Prescription Drug Monitoring Program (PDMP) and for female participants; a negative pregnancy test result was required. Participants were then randomized to one of two posoperative analgesic treatment groups—nonopioid or opioid—and received a blinded study medication kit corresponding to their assigned group.

For up to 9 days postsurgery (±5 days), participants were instructed to manage their pain using the assigned study medication and to complete electronic diaries twice daily. These electronic diaries captured data on pain intensity, pain interference, sleep quality, side effects, and any use of rescue analgesics (5 mg oxycodone).

At the postoperative follow-up appointment (visit 2), participants underwent a clinical examination and recorded their pain experience, pain interference, sleep quality, side effects experienced in the last 24 hours, and their overall satisfaction with the assigned medication.

### DATA ANALYSIS

All relevant study measures including baseline demographics, preoperative pain measures, intra-operative measures, and postoperative outcomes were summarized using frequency and percentage for categorical variables and means with standard deviations for continuous data. Bivariate testing was performed using Pearson’s χ^2^ or Fisher’s exact tests for categorical measures, and independent sample *t* tests were implemented to compare continuous measures. In bivariate testing, we tested differences between the steroid and no steroid group for each of the opioid and nonopioid groups, as well as tested the differences between the opioid and nonopioid groups for each of the steroid and no steroid groups; to account for these multiple comparison, a Bonferroni adjustment was applied (*P* value < .05/2 = 0.025). For postoperative pain, which was assessed at 4 different time points, a Bonferroni adjustment was also applied to correct for multiple comparisons (*P* value < .05/4 = 0.0125). For secondary outcomes which were only collected at the postoperative visit, a *P* value < .05 was used to assess statistical significance.

Similar to the statistical methods used in the primary OARS trial analyses to test differences in postoperative pain across steroid use and analgesic treatment groups, we used mixed-effects regression models that were used where pain score was modeled as a function of steroid use (steroid vs no steroid), analgesic treatment (non-opioid vs Opioid), day, and day by analgesic by steroid use interactions as fixed effect independent variables, and participants and clinical sites as random effects. Linear contrasts were constructed to compare the mean differences (analgesic effect) between opioids and nonopioids for steroid exposure separately and to compare mean differences (steroid effect) between steroids and no steroids for analgesic groups separately as well. Confidence intervals for means were calculated based on a *P* value < .05/2 = 0.025. Mean differences across groups were assessed at 4 time points (day 1, day 2, day 3, and the entire post-operative period), using a 99.375% confidence interval (*P* value < .00625), after the Bonferroni adjustment.

Secondary outcomes: Mixed-effects regression techniques were used to assess differences in satisfaction with pain medication and number of unused analgesia tablets returned across steroid and analgesic treatment groups using methods similar to those described for pain. However, as these outcomes were measured at a single time point, time was not included as a covariate in the model. The need for emergency clinic visit, rescue medication, and the occurrence of side effects were modeled using random effects logistic regression techniques with steroid use, analgesic treatment, and analgesic treatment by steroid use interactions as fixed effect independent variables and clinical site as a random effect. Statistical significance was defined using a *P* value < .05/2 = 0.025 for each outcome.

All regression models were adjusted for demographic variables, preoperative pain measures, and intra-operative approaches to account for baseline difference between the steroid and no steroid groups. Statistical analyses were performed using SAS version 9.4 (SAS Institute).

## Results

A total of 1,774 subjects from the 5 participating sites of Chicago (n = 428 (24.1%)), Maryland (275 (15.5%)), Michigan (266 (15.0%)), Rochester (429 (24.2%)), and Rutgers (376 (21.2%)) met the inclusion criteria for this study. Of those, 513 (28.9%) subjects were administered intra-operative IV dexamethasone. The mean (SD) age of participants was 25.7 (6.2) years, 893 (50.3%) were female, 555 (31.3%) were Hispanic, 498 (28.1%) were Black, and 339 (19.1%) were White. Most participants did not have a history of smoking, did not present with swelling, and had a low level of preoperative pain. Mean (SD) surgical duration was 38.7 (19.8) minutes with a mean of 2.7 (1.2) teeth extracted, 853 (48.1%) of participants had a full bony impaction and 1,379 (77.7%) had the most difficult surgical technique (osteotomy and sectioning) (Table 2).

Compared to participants who did not receive intra-operative IV steroids, those who did were on average younger, less likely to smoke, had less preoperative swelling, and less preoperative pain. There were also racial differences between the groups; the proportion of Hispanics in the steroid group was twice that of the nonsteroid group (48 vs 24%). Participants in the steroid group were more likely to have a full bony impaction and receive general anesthesia, antibiotics, and Marcaine (Table 2).

A comprehensive analysis of the covariates in Table 2 was then performed for the 4 sub-groups represented by the interaction between analgesic treatment and steroid use: *1)* nonopioid + no steroid: 626 (35.3%), *2)* opioid + no steroid: 635 (35.8%), *3)* nonopioid + steroid: 265 (14.9%), and *4)* opioid + steroid: 248 (14.0%). Significant differences were observed across all 4 groups for most covariates. When analgesic treatment was held constant and steroid exposure varied, statistically significant differences were observed in baseline participant characteristics between the groups. On the other hand, when steroid exposure was held constant and analgesic treatment varied, the baseline participant characteristics were not statistically different (Table 3).

Average postoperative pain, the primary outcome, was first analyzed in a univariate fashion for each covariate on day 1, day 2, day 3, and the entire postoperative period, separately. On day 1, males reported a significantly lower mean pain (SD) rating of 1.3 (1.9) than females (1.8 (2.2), *P* < .001), a finding that was echoed across the other time points as well. Postoperative pain was weakly, positively correlated with age (r = 0.14), preoperative pain (r = 0.19), and surgical duration (r = 0.13) (*P* < .001). Participants with preoperative swelling, at least one full bony impaction, and those whose surgeries required more difficult surgical techniques (osteotomy or osteotomy and tooth sectioning) also reported higher levels of postoperative pain. In the bivariate analysis, intra-operative antibiotic and Marcaine use, which occurred based on operator preference, were associated with lower pain ratings. The results were similar across all time points (Table 4).

Tables 5 and 6 present the estimates from a multivariable mixed-effects regression model, which tested the interaction of randomly assigned analgesic treatment, intra-operative steroid use, and time on participant’s postoperative pain levels while controlling for both preoperative and operative covariates. On postoperative day 1, the 4 sub-groups: (1) nonopioid + no steroid, (2) opioid + no steroid, (3) nonopioid + steroid, and (4) opioid + steroid, had the following mean (97.5% CI) pain values: 3.78 (3.45, 4.10), 4.57 (4.24, 4.90), 3.00 (2.61, 3.39), and 3.35 (2.94, 3.75), respectively. When focusing solely on participants who were randomized to nonopioid analgesics, there was a significant mean difference in postoperative day 1 pain between the steroid exposure sub-groups. The mean (99.375% CI) difference was 0.78 (0.25, 1.30) with the nonopioid + steroid group reporting less pain than the nonopioid + no steroid group (*P* < .001). Likewise, when examining the participants who were randomized to receive opioids, there was a significant mean difference in postoperative day 1 pain of 1.22 (0.68, 1.77) with the sub-group who were given steroids reporting less pain (*P* < .001) (Table 5).

For postoperative day 1, there was a significant mean difference (99.375% CI) of −0.80 (−1.12, −0.47) between the nonopioid and opioid treatment groups who both had no steroid exposure (*P* < .001), indicating that these participants experienced significantly less pain when taking nonopioid alone versus opioids alone. On the other hand, for the sub-groups who received steroids, there was no significant mean difference (−0.35 (−0.86, 0.17)) between the nonopioid and opioid treatment arms (*P* = .2). While the use of intra-operative Marcaine was associated with lower postoperative pain in bivariate analysis, there was no independent relationship in the multivariate analysis (Table 6).

On postoperative days 2 and 3, there were no significant mean differences between any of the comparison groups (Tables 5 and 6).

Across the full postoperative follow-up period, the 4 sub-groups: *1)* nonopioid + no steroid, *2)* opioid + no steroid, *3)* nonopioid + steroid, and *4)* opioid + steroid showed a mean (97.5% CI) postoperative pain score of 2.57 (2.24, 2.89), 2.80 (2.47, 3.13), 2.02 (1.58, 2.45), and 2.42 (1.99, 2.86), respectively. The mean difference (99.375% CI) between those who did and did not receive steroids in the nonopioid treatment group was 0.55 (−0.04, 1.14) (*P* = .026), indicating that steroids reduced average postoperative pain across all days. However, the same effect was not seen for those who were randomized to the opioid treatment arm (Tables 5 and 6).

Adverse outcomes were recorded in the OARS trial as the following: side effects reported in electronic diary, emergency clinic visit required, and postoperative rescue medication prescribed. Among the participants randomized to the nonopioid treatment arm, the odds ratios for the recorded adverse outcomes based on steroid exposure ranged from 0.98 to 1.01 with no significant *P* values. Similarly, for those who were randomized to the opioid arm, the odds ratios based on steroid exposure ranged from 0.99 to 1.02 with no significant differences being detected. In summary, the addition of steroids did not increase the likelihood of having an adverse outcome (Table 7).

## Discussion

Traditionally, corticosteroids have been administered preoperatively to reduce postoperative trismus and swelling for third molar surgery. ^5,9,20^ A systematic review of 27 articles by Sugragan et al concluded that corticosteroids provide optimal postoperative pain relief when given during the preoperative phase. ^10^ However, the results of this secondary cohort study demonstrate that IV corticosteroids, given intra-operatively, provide significant pain reduction for the first 24 hours after surgery. This is particularly relevant, as previous research has shown that postoperative pain from third molar surgery peaks within the first 24 hours, with multiple sources specifying this occurring within the first 6 to 12 hours. ^5,21–23^ The findings of this study indicate that the analgesic effects of intra-operative IV corticosteroids are well-timed to coincide with this peak period of postoperative pain, supporting their clinical utility. While corticosteroids are well known for reducing postoperative swelling, these results further emphasize their role in postoperative pain management, even when administered during the intra-operative period.

The secondary aim of this paper was to evaluate whether combining intra-operative IV corticosteroids with either postoperative nonopioids or nonopioids opioids resulted in a superior outcome and to determine which combination was more effective. A multivariate model comparing the 4 analytic sub-groups revealed that on postoperative day 1, any combination that included steroids resulted in significantly decreased postoperative pain. Notably, the steroid + nonopioid group exhibited the lowest average postoperative pain across all measured days. Regardless of steroid exposure, the nonopioid treatment arms consistently had lower mean pain scores than their opioid counterparts across days 1, 2, 3, and overall. Across the entire postoperative follow-up period, the use of steroids with nonopioids was associated with a mean (99.375% CI) pain reduction of 0.55 (−0.04, 1.14) (*P* = .026). In summary, multivariate analysis indicates that intra-operative IV corticosteroids are most effective on postoperative day 1 regardless of postoperative analgesic and provide a pain-relieving effect across all days when combined with postoperative nonopioids. Given the adverse effects associated with opioid use, these findings support the use of intra-operative IV steroids in combination with postoperative nonopioidsanalgesics.

Adverse outcomes are also an important consideration when evaluating the inclusion of intra-operative IV corticosteroids in any pain management protocols. The OARS study recorded the following adverse outcomes: side effects reported in electronic diary, whether or not an emergency clinic visit was required, and whether or not postoperative rescue medication was prescribed. When examining these outcomes among all 4 analytic sub-groups, the incidence of adverse events was not significantly different, suggesting that the use of IV dexamethasone did not increase the risk of unwanted side effects. Unsurprisingly, when looking at the participants who were not given a steroid, the nonopioid arm had a 5% less likely chance of reporting a side effect in their electronic diary and a 3% less likely chance of requiring an emergency clinic visit. In short, it appears that adding steroids did not increase the risk of unwanted side effects, and the use of postoperative nonopioids alone proved to be safer than the use of postoperative opioids (Table 7).

Currently, there are few studies researching the combination of corticosteroids when paired with either nonopioid analgesics or opioids for postoperative pain management. A systematic review by Momesso et al demonstrated that for third molar surgery, the combination of steroids and NSAIDs was superior to either medication alone when assessing pain for the first 48 postoperative hours.^24^ Braxendale et al in a study of 50 adult patients concluded that dexamethasone 8 mg given 2 hours preoperatively reduced the need for opioids to be given postoperatively. However, the study did not assess any further than 16 hours postprocedure. ^11^ When attempting to evaluate the effectiveness of corticosteroids and opioids in managing third molar postoperative pain, no data could be found. Assessing studies from other medical specialties, Mercadante et al concluded that among cancer patients, corticosteroids did not improve opioid analgesia or reduce opioid consumption. ^25^ In patients who have received a lumbar discectomy, Aminmansour et al demonstrated that a single preoperative high dose of dexamethasone had reduced postoperative pain and opioid usage. ^26^ With the current opioid epidemic claiming an estimated 81,000 lives per year, the current pain management technique should be to limit opioid usage. ^27^

The strength of this paper lies in the total number of participants with data collected at 5 separate locations, resulting in a greater generalizability of these results. Furthermore, unlike other pain studies that track postoperative pain for only a few hours, these participants were followed for a period of 1 to 2 weeks, resulting in more opportunities to ask about postoperative pain over time and a longer timeframe to observe whether adverse outcomes would occur. The main limitation of this paper is that it is a secondary study to the OARS trial. Although adverse events were addressed in the OARS trial, the ability to assess specific side effects caused by the corticosteroids was limited. However, based on multiple studies, a one-time dose of 4 to 8 mg of dexamethasone is safe with minimal side effects for ASA I and II patients. ^28–30^

This study demonstrates that intra-operative IV dexamethasone significantly reduces postoperative pain during the first 24 hours following third molar surgery. Notably, in those receiving corticosteroid, pain relief was observed in both the opioid and nonopioid groups on day 1, with a sustained analgesic effect across the entire postoperative follow up period in the nonopioid group. These findings support the incorporation of intra-operative IV dexamethasone alongside postoperative NSAIDs and acetaminophen as part of a multimodal approach to pain management, potentially reducing the need for opioid prescriptions in routine third molar surgery.

## Figures and Tables

**Table 1.** INCLUSION AND EXCLUSION CRITERIA

Inclusion Criteria	Exclusion Criteria

	
Undergoing extraction of one or more partial or fully impacted mandibular third molars.	History of gastrointestinal bleeding and/or peptic ulcer.
Able to understand the informed consent process and provide signed and dated informed consent.	History of renal disease (excluding kidney stones).
Willing to comply with all study procedures and be available for the duration of the study.	History of hepatic disease.
Aged 18 yr or older.	History of cardiovascular disease (myocardial infarction or stroke within the past 6 mo).
In good general health as evidenced by medical history.	History of bleeding disorder.
Able to take ibuprofen, acetaminophen (paracetamol), and hydrocodone.	History of respiratory depression.
If female, must agree to use one of the following methods of contraception while participating in the study: contraceptive pill, intrauterine device (IUD), condoms, or abstinence.	Any prior respiratory effect of an opioid or other anesthetic drug that required respiratory support postoperatively.
Must have a negative pregnancy test if female	Active or untreated asthma.
	History of known allergic reactions to ibuprofen, acetaminophen, hydrocodone, or anesthesia.
	Currently taking any of the following medications: • CYP3A4 inhibitors, such as macrolide antibiotics (eg, erythromycin), azole antifungal agents (eg, ketoconazole), and protease inhibitors (eg, ritonavir), which may increase plasma concentrations of hydrocodone bitartrate and acetaminophen and prolong opioid adverse reactions, potentially causing fatal respiratory depression. central nervous system depressants, including benzodiazepines (eg, Valium, Xanax, and Klonopin).
	Consuming three or more alcoholic drinks daily and/or having a history of alcoholism.
	History of drug or alcohol abuse.
	Family history of drug or alcohol abuse in a first-degree relative.
	Having had one or more opioid prescriptions filled within the past 6 mo.
	Currently pregnant or lactating.
	Prior participation in this study.
	Use of intra-operative bupivacaine or any other long-acting local anesthetic.
	Use of intra-operative anti-inflammatory agents given orally. Oral corticosteroids given at the time of surgery.

Look et al. Intra-OPERATIVE Dexamethasone with Opioids or Non-Opioids? J Oral Maxillofac Surg 2026.

**Table 2.** BASELINE CHARACTERISTICS AND SURGICAL PARAMETERS BY INTRA-OPERATIVE IV STEROID USE

		Intra-Operative IV Steroid		
Baseline Characteristic	All (n = 1,774)	No (n = 1,261)	Yes (n = 513)	*P* Value

Age, mean (SD)*	25.7 (6.2)	26.2 (6.3)	24.4 (5.8)	<.0001
Female	893 (50.3)	617 (48.9)	276 (53.8)	.063
Race/ethnicity				<.0001
Non-Hispanic Black	498 (28.1)	361 (28.6)	137 (26.7)	
Non-Hispanic White	339 (19.1)	281 (22.3)	58 (11.3)	
Non-Hispanic Asian	271 (15.3)	218 (17.3)	53 (10.3)	
Hispanic	555 (31.3)	308 (24.4)	247 (48.1)	
Non-Hispanic Other	62 (3.49)	54 (4.28)	8 (1.56)	
Do not want to report	49 (2.76)	39 (3.09)	10 (1.95)	
Site				<.0001
Chicago	428 (24.1)	262 (20.8)	166 (32.4)	
Maryland	275 (15.5)	214 (17.0)	61 (11.9)	
Michigan	266 (15.0)	246 (19.5)	20 (3.90)	
Rochester	429 (24.2)	429 (34.0)	0 (0.00)	
Rutgers	376 (21.2)	110 (8.72)	266 (51.9)	
Education				<.0001
Some high school	97 (5.47)	59 (4.68)	38 (7.41)	
Graduated high school	969 (54.6)	656 (52.0)	313 (61.0)	
Associate degree	191 (10.8)	137 (10.9)	54 (10.5)	
Bachelor’s degree	347 (19.6)	261 (20.7)	86 (16.8)	
Master’s degree	103 (5.81)	90 (7.14)	13 (2.53)	
Doctoral degree	43 (2.42)	37 (2.93)	6 (1.17)	
Do not want to report	24 (1.35)	21 (1.67)	3 (0.58)	
Smoking history				.007
Don’t smoke at all	1,560 (87.9)	1,088 (86.3)	472 (92.0)	
Smoke less than 1 pack a day	190 (10.7)	152 (12.1)	38 (7.41)	
Smoke 1 pack per day	20 (1.13)	17 (1.35)	3 (0.58)	
Smoke > 1 pack per day	4 (0.23)	4 (0.32)	0 (0.00)	
Preoperative swelling				<.0001
Without swelling	1,484 (83.7)	1,018 (80.7)	466 (90.8)	
Mild swelling	223 (12.6)	183 (14.5)	40 (7.80)	
Moderate swelling	51 (2.87)	44 (3.49)	7 (1.36)	
Severe swelling	16 (0.90)	16 (1.27)	0 (0.00)	
Worst pain, mean (SD)*	1.6 (2.8)	1.9 (3.0)	0.9 (2.2)	<.0001
Average pain, mean (SD)*	1.2 (2.3)	1.4 (2.4)	0.7 (1.8)	<.0001
Pain tolerance, mean (SD)*	5.9 (2.3)	6.0 (2.3)	5.7 (2.3)	.026

Surgical parameters				

Minutes in surgery, mean (SD)*	38.7 (19.8)	39.2 (19.8)	37.5 (19.7)	.1
Total teeth extracted, mean (SD)*	2.7 (1.2)	2.7 (1.2)	2.8 (1.2)	.8
Maximum impaction level				.001
Partial bony impaction	921 (51.9)	740 (58.7)	181 (35.3)	
Full bony impaction	853 (48.1)	521 (41.3)	332 (64.7)	
Anesthesia type				<.0001
Local only	860 (48.5)	850 (67.4)	10 (1.95)	
Oral/nitrous/conscious sedation	256 (14.4)	220 (17.4)	36 (7.02)	
General	658 (37.1)	191 (15.1)	467 (91.0)	
Intra-operative antibiotic used	279 (15.7)	47 (3.73)	232 (45.2)	<.0001
Intra-operative Marcaine used	134 (7.55)	16 (1.27)	118 (23.0)	<.0001
Most difficult surgical technique				<.0001
Forceps or elevator only	100 (5.64)	79 (6.26)	21 (4.09)	
Osteotomy	295 (16.6)	246 (19.5)	49 (9.55)	
Osteotomy and tooth sectioning	1,379 (77.7)	936 (74.2)	443 (86.4)	

* Statistics reported are frequency and percent unless otherwise noted as mean and standard deviation.

Look et al. Intra-OPERATIVE Dexamethasone with Opioids or Non-Opioids? J Oral Maxillofac Surg 2026.

**Table 3.** BASELINE CHARACTERISTICS AND SURGICAL PARAMETERS BY INTRA-OPERATIVE IV STEROID USE AND ANALGESIC TREATMENT

	No Steroid Groups		IV Steroid Groups		All Groups	*P* Value: Tests Between/Across Groups			
	Nonopioid	Opioid	Nonopioid	Opioid		Steroid vs No Steroid		Nonopioid vs Opioid	
Demographic Characteristics	1	2	3	4	1 vs 2 vs 3 vs 4	Nonopioid	Opioid	No Steroid	Steroid
	n (%)	n (%)	n (%)	n (%)		1 vs 3	2 vs 4	1 vs 2	3 v 4

Sample size	626 (35.3)	635 (35.8)	265 (14.9)	248 (14.9)					
Age, mean (SD)*	26.1 (5.9)*	26.4 (6,7)*	24.2 (5.8)*	24.5 (5.9)*	<.0001	<0.001	0.000	0.9	0.9
Female	303 (48.4)	314 (49.4)	147 (55.5)	129 (52.0)	0.2	0.1	0.9	0.9	0.9
Race/ethnicity					<.0001	<0.001	<0.001	0.9	0.9
Non-Hispanic Black	181 (28.9)	180 (28.3)	69 (26.0)	68 (27.4)					
Non-Hispanic White	137 (21.9)	144 (22.7)	33 (12.5)	25 (10.1)					
Non-Hispanic Asian	110 (17.6)	108 (17.0)	30 (11.3)	23 (9.27)					
Hispanic	152 (24.3)	156 (24.6)	125 (47.2)	122 (49.2)					
Non-Hispanic Other	28 (4.47)	26 (4.09)	4 (1.51)	4 (1.61)					
Do not want to report	18 (2.88)	21 (3.31)	4 (1.51)	6 (2.42)					
Site					<.0001	<0.001	<0.001	0.9	<0.001
Chicago	129 (20.6)	133 (20.9)	86 (32.5)	80 (32.3)					
Maryland	103 (16.5)	111 (17.5)	35 (13.2)	26 (10.5)					
Michigan	125 (20.0)	121 (19.1)	11 (4.15)	9 (3.63)					
Rochester	211 (33.7)	218 (34.3)	0 (0.00)	0 (0.00)					
Rutgers	58 (9.27)	52 (8.19)	133 (50.2)	133 (53.6)					
Education					0.002	0.011	0.018	0.9	0.9
Some high school	24 (3.83)	35 (5.51)	17 (6.42)	21 (8.47)					
Graduated high school	319 (51.0)	337 (53.1)	158 (59.6)	155 (62.5)					
Associate degree	71 (11.3)	66 (10.4)	32 (12.1)	22 (8.87)					
Bachelor’s degree	135 (21.6)	126 (19.8)	46 (17.4)	40 (16.1)					
Master’s degree	49 (7.83)	41 (6.46)	7 (2.64)	6 (2.42)					
Doctoral degree	18 (2.88)	19 (2.99)	3 (1.13)	3 (1.21)					
Do not want to report	10 (1.60)	11 (1.73)	2 (0.75)	1 (0.40)					
Smoking history					0.1	0.2	0.1	0.9	<0.001
Don’t smoke at all	541 (86.4)	547 (86.1)	243 (91.7)	229 (92.3)					
Smoke less than 1 pack a day	72 (11.5)	80 (12.6)	21 (7.92)	17 (6.85)					
Smoke 1 pack per day	11 (1.76)	6 (0.94)	1 (0.38)	2 (0.81)					
Smoke > 1 pack per day	2 (0.32)	2 (0.31)	0 (0.00)	0 (0.00)					
Preoperative Swelling					<.0001	0.1	<0.001	0.5	<0.001
Without swelling	519 (82.9)	499 (78.6)	239 (90.2)	227 (91.5)					
Mild swelling	81 (12.9)	102 (16.1)	21 (7.92)	19 (7.66)					
Moderate swelling	20 (3.19)	24 (3.78)	5 (1.89)	2 (0.81)					
Severe swelling	6 (0.96)	10 (1.57)	0 (0.00)	0 (0.00)					
Worst pain, mean (SD)*	1.9 (3.0)*	1.9 (3.0)*	0.9 (2.1)*	1.0 (2.3)*	<.001	<0.001	<0.001	0.9	0.9
Average pain, mean (SD)*	1.4 (2.3)*	1.4 (2.5)*	0.6 (1.7)*	0.7 (1.8)*	<.001	<0.001	<0.001	0.9	0.9
Pain tolerance, mean (SD)*	6.0 (2.3)*	6.0 (2.3)*	5.8 (2.2)*	5.6 (2.4)*	0.2	0.8	0.0	0.9	0.4

Surgical parameters									

Minutes in surgery, mean (SD)*	38.8 (19.4)*	39.6 (20.1)*	37.4 (20.1)*	37.5 (19.4)*	0.2	0.7	0.3	0.9	0.9
Total teeth extracted, mean (SD)*	2.7 (1.2)*	2.8 (1.5)*	2.9 (1.2)*	2.7 (1.2)*	0.2	0.2	0.4	0.6	0.2
Maximum impaction level					<.0001	<0.001	<0.001	0.8	0.9
Partial bony impaction	375 (59.9)	365 (57.5)	93 (35.1)	88 (35.5)					
Full bony impaction	251 (40.1)	270 (42.5)	172 (64.9)	160 (64.5)					
Anesthesia type					<.0001	<0.001	<0.001	0.9	1.0
Local only	423 (67.6)	427 (67.2)	5 (1.89)	5 (2.02)					
Oral/nitrous/conscious sedation	106 (16.9)	114 (18.0)	22 (8.30)	14 (5.65)					
General	97 (15.5)	94 (14.8)	238 (89.8)	229 (92.3)					
Intra-operative antibiotic used	24 (3.83)	23 (3.62)	114 (43.0)	118 (47.6)	<.0001	<0.001	<0.001	0.9	0.6
Intra-operative opioid used	102 (16.3)	113 (17.8)	197 (74.3)	186 (75.0)	<.0001	<0.001	<0.001	0.9	0.9
Intra-operative Marcaine used	9 (1.44)	7 (1.10)	63 (23.8)	55 (22.2)	<.0001	<0.001	<0.001	0.9	0.9
Most difficult surgical technique					<.0001	<0.001	0.001	0.8	0.9
Forceps or elevator only	44 (7.03)	35 (5.51)	13 (4.91)	8 (3.23)					
Osteotomy	126 (20.1)	120 (18.9)	26 (9.81)	23 (9.27)					
Osteotomy and tooth sectioning	456 (72.8)	480 (75.6)	226 (85.3)	217 (87.5)					

* Statistics reported are frequency (%) unless otherwise noted as mean and standard deviation (SD).

Look et al. Intra-OPERATIVE Dexamethasone with Opioids or Non-Opioids? J Oral Maxillofac Surg 2026.

**Table 4.** BASELINE CHARACTERISTICS AND SURGICAL PARAMETERS BY POSTOPERATIVE PAIN

	Average Postoperative Pain							
	Day 1	Adjusted *P* Value^†^	Day 2	Adjusted *P* Value^†^	Day 3	Adjusted *P* Value^†^	All Days	Adjusted *P* Value
	Mean (SD)		Mean (SD)		Mean (SD)		Mean (SD)	

Age*	0.14*	<.001	0.14*	<.001	0.14*	<.001	0.14*	<.001
Sex		<.001		<.001		<.001		<.001
Female	1.8 (2.2)		1.8 (2.2)		1.8 (2.2)		1.7 (2.2)	
Male	1.3 (1.9)		1.3 (1.9)		1.3 (1.9)		1.2 (1.8)	
Race/ethnicity		<.001		<.001		<.001		<.001
Non-Hispanic Black	1.9 (2.3)		1.9 (2.4)		1.9 (2.4)		1.8 (2.3)	
Non-Hispanic White	1.6 (1.9)		1.6 (1.9)		1.6 (1.9)		1.6 (1.9)	
Non-Hispanic Asian	1.0 (1.5)		1.0 (1.4)		1.0 (1.4)		1.0 (1.4)	
Hispanic	1.4 (2.1)		1.4 (2.1)		1.4 (2.1)		1.4 (2.0)	
Non-Hispanic other	1.9 (2.2)		1.9 (2.2)		1.9 (2.2)		1.8 (2.2)	
Do not want to report	1.6 (2.5)		1.5 (2.5)		1.4 (2.2)		1.4 (2.4)	
Site		<.001		<.001		<.001		<.001
Chicago	1.5 (2.1)		1.6 (2.2)		1.5 (2.1)		1.5 (2.1)	
Maryland	2.2 (2.4)		2.2 (2.4)		2.2 (2.4)		2.1 (2.3)	
Michigan	1.5 (1.9)		1.5 (1.9)		1.6 (1.9)		1.5 (1.9)	
Rochester	1.2 (1.9)		1.2 (1.9)		1.2 (1.9)		1.2 (1.9)	
Rutgers	1.3 (2.0)		1.4 (2.0)		1.3 (2.0)		1.3 (1.9)	
Education		.003		.002		.004		<.001
Some high school	1.4 (2.1)		1.4 (2.2)		1.4 (2.2)		1.4 (2.1)	
Graduated high school	1.6 (2.2)		1.6 (2.2)		1.6 (2.2)		1.5 (2.2)	
Associate degree	1.9 (2.1)		1.9 (2.2)		1.9 (2.2)		1.8 (2.1)	
Bachelor’s degree	1.5 (1.8)		1.4 (1.8)		1.4 (1.8)		1.4 (1.8)	
Master’s degree	1.2 (1.5)		1.2 (1.6)		1.2 (1.6)		1.2 (1.6)	
Doctoral degree	1.2 (1.6)		1.2 (1.6)		1.2 (1.6)		1.1 (1.6)	
Do not want to report	1.2 (1.9)		1.3 (1.9)		1.3 (1.9)		1.1 (1.7)	
Smoking history		<.001		<.001		<.001		<.001
Don’t smoke at all	1.4 (2.0)		1.4 (2.0)		1.4 (2.0)		1.4 (2.0)	
Smoke less than 1 pack a day	2.4 (2.7)		2.3 (2.6)		2.3 (2.6)		2.2 (2.5)	
Smoke 1 pack per day	1.2 (1.6)		1.6 (2.2)		1.6 (2.2)		1.6 (2.2)	
Smoke >1 pack per day	1.0 (1.3)		1.0 (1.3)		1.0 (1.3)		1.0 (1.2)	
Preoperative Swelling		<.001		<.001		<.001		<.001
Without swelling	1.4 (2.0)		1.4 (2.0)		1.4 (2.0)		1.4 (1.9)	
Mild swelling	1.9 (2.3)		1.9 (2.3)		1.9 (2.3)		1.8 (2.3)	
Moderate swelling	2.8 (2.7)		2.8 (2.7)		2.8 (2.7)		2.7 (2.5)	
Severe swelling	2.7 (3.0)		2.9 (3.0)		2.3 (2.3)		2.6 (2.9)	
Worst pain*	0.19*	<.001	0.18*	<.001	0.18*	<.001	0.17*	<.001
Average Pain*	0.19*	<.001	0.19*	<.001	0.19*	<.001	0.18*	<.001
Pain tolerance*	−0.01*	.7	−0.01*	.7	0.00*	.9	0.00*	.7
Surgical parameters								
Minutes in surgery*	0.13*	<.001	0.12*	<.001	0.12*	<.001	0.12*	<.001
Total teeth extracted*	−0.01*	.6	0.00*	.9	0.00*	1.0	0.00*	1.0
Maximum impaction level		<.001		<.001		<.001		.6
Partial bony impaction	1.4 (2.0)		1.4 (2.0)		1.4 (2.0)		1.4 (2.0)	
Full bony impaction	1.7 (2.1)		1.7 (2.2)		1.7 (2.1)		1.6 (2.1)	
Anesthesia type		.002		.002		.001		<.001
Local only	1.4 (2.1)		1.4 (2.1)		1.4 (2.1)		1.4 (2.0)	
Oral/nitrous/conscious sedation	1.8 (2.0)		1.8 (2.1)		1.8 (2.1)		1.7 (2.0)	
General	1.5 (2.1)		1.6 (2.1)		1.5 (2.1)		1.5 (2.0)	
Intra-operative antibiotic used		.012		.027		.024		<.001
No	1.6 (2.1)		1.6 (2.1)		1.6 (2.1)		1.5 (2.1)	
Yes	1.3 (1.9)		1.4 (2.0)		1.3 (1.9)		1.3 (1.9)	
Intra-operative Marcaine used		.012		.3		.038		<.001
No	1.6 (2.1)		1.6 (2.1)		1.5 (2.1)		1.5 (2.0)	
Yes	1.2 (1.9)		1.3 (1.9)		1.3 (1.9)		1.2 (1.9)	
Most difficult surgical technique		.003		.003		.003		<.001
Forceps or elevator only	1.2 (2.1)		1.2 (2.1)		1.2 (2.1)		1.2 (2.0)	
Osteotomy	1.3 (2.0)		1.3 (2.1)		1.3 (2.1)		1.3 (2.0)	
Osteotomy and tooth sectioning	1.6 (2.1)		1.6 (2.1)		1.6 (2.1)		1.6 (2.0)	

* For the continuous variables (age, worst/average preoperative pain, pain tolerance, minutes in surgery, and number of teeth extracted), the statistic reported is the Pearson correlation Rho (r) and the associated *P* value. For all other variables, the mean (standard deviation) is reported.

† *P* values have been adjusted using a Bonferroni correction to account for the multiple comparisons over time (*P* value = .05/4 = 0.0125).

Look et al. Intra-OPERATIVE Dexamethasone with Opioids or Non-Opioids? J Oral Maxillofac Surg 2026.

**Table 5.** MIXED EFFECTS MODEL OF AVERAGE POSTOPERATIVE PAIN BY IV INTRA-OPERATIVE STEROID USE AND ANALGESIC TREATMENT (STEROID VS NO STEROID HOLDING ANALGESIC TREATMENT CONSTANT)

	Nonopioid Groups			
	Nonopioid: No Steroid*	Nonopioid : Steroid*	Mean Difference: No Steroid vs Steroid	
Time	Mean (97.5% CI)	Mean (97.5% CI)	Difference (99.375% CI)	Adjusted *P* Value^†^

				
Day 1 am/pm	3.78 (3.45, 4.10)	3.00 (2.61, 3.39)	0.78 (0.25, 1.30)	<.001
Day 2 am/pm	3.03 (2.70, 3.35)	2.82 (2.43, 3.21)	0.21 (−0.32, 0.74)	1.0
Day 3 am/pm	2.77 (2.44, 3.10)	2.73 (2.34, 3.12)	0.04 (−0.49, 0.57)	1.0
Entire postoperative	2.57 (2.24, 2.89)	2.02 (1.58, 2.45)	0.55 (−0.04, 1.14)	.026
	Opioid Groups			
	Opioid: No Steroid*	Opioid: Steroid*	Mean Difference: No Steroid vs Steroid	
Time	Mean (97.5% CI)	Mean (97.5% CI)	Difference (99.375% CI)	Adjusted *P* Value^†^

				
Day 1 am/pm	4.57 (4.24, 4.90)	3.35 (2.94, 3.75)	1.22 (0.68, 1.77)	<.001
Day 2 am/pm	3.34 (3.01, 3.67)	2.99 (2.58, 3.39)	0.35 (−0.19, 0.89)	.2
Day 3 am/pm	2.85 (2.53, 3.18)	2.77 (2.36, 3.17)	0.09 (−0.46, 0.63)	1.0
Entire postoperative	2.80 (2.47, 3.13)	2.42 (1.99, 2.86)	0.37 (−0.21, 0.95)	.2

* Groups listed in the order they are compared; for example, nonopioid versus opioid is calculated: nonopioid -opioid.

† *P* values have been adjusted using a Bonferroni correction to account for the multiple comparisons over time (alpha = 0.025/4 = 0.00625).

Look et al. Intra-OPERATIVE Dexamethasone with Opioids or Non-Opioids? J Oral Maxillofac Surg 2026.

**Table 6.** MIXED EFFECTS MODEL OF AVERAGE POSTOPERATIVE PAIN BY IV INTRA-OPERATIVE STEROID USE AND ANALGESIC TREATMENT (NONOPIOID VS OPIOID HOLDING STEROID TREATMENT CONSTANT)

	No Steroid Groups			
	No Steroid: Nonopioid*	No Steroid: Opioid*	Mean Difference: Nonopioid vs Opioid	
Time	Mean (97.5% CI)	Mean (97.5% CI)	Difference (99.375% CI)	Adjusted *P* Value^†^


Day 1 am/pm	3.78 (3.45, 4.10)	4.57 (4.24, 4.90)	−0.80 (−1.12, −0.47)	<.001
Day 2 am/pm	3.03 (2.70, 3.35)	3.34 (3.01, 3.67)	−0.31 (−0.64, 0.02)	.025
Day 3 am/pm	2.77 (2.44, 3.10)	2.85 (2.53, 3.18)	−0.09 (−0.42, 0.24)	1.0
Entire postoperative	2.57 (2.24, 2.89)	2.80 (2.47, 3.13)	−0.23 (−0.56, 0.10)	.2
	Steroid Groups			
	Steroid: Nonopioid*	Steroid: Opioid*	Mean Difference: Nonopioid vs. Opioid	
Time	Mean (97.5% CI)	Mean (97.5% CI)	Difference (99.375% CI)	Adjusted *P*-Value^†^


Day 1 am/pm	3.00 (2.61, 3.39)	3.35 (2.94, 3.75)	−0.35 (−0.86, 0.17)	.2
Day 2 am/pm	2.82 (2.43, 3.21)	2.99 (2.58, 3.39)	−0.17 (−0.69, 0.35)	1.0
Day 3 am/pm	2.73 (2.34, 3.12)	2.77 (2.36, 3.17)	−0.04 (−0.56, 0.48)	1.0
Entire postoperative	2.02 (1.58, 2.45)	2.42 (1.99, 2.86)	−0.41 (−1.02, 0.21)	.2

*Note:* Mixed effects models adjusted for age, sex, race/ethnicity, average pain at preoperative visit, number of teeth extracted, maximum impaction level, method used to extract teeth, type of anesthesia administered, and use of intra-operative antibiotics and intra-operative Marcaine. Average pain values ranged from 0 to 10. Numeric Rating Scale: 0 = no pain to 10 = worst pain imaginable.

* Groups listed in the order they are compared; for example, nonopioid versus opioid is calculated: nonopioid -opioid.

† *P* values have been adjusted using a Bonferroni correction to account for the multiple comparisons over time (alpha = 0.025/4 = 0.00625).

Look et al. Intra-OPERATIVE Dexamethasone with Opioids or Non-Opioids? J Oral Maxillofac Surg 2026.

**Table 7.** ESTIMATES FROM REGRESSION MODELS FOR SECONDARY OUTCOMES BY IV INTRA-OPERATIVE STEROID USE AND ANALGESIC TREATMENT GROUPS (STEROID VS NO STEROID HOLDING ANALGESIC TREATMENT CONSTANT)

	Nonopioid Groups			
	Nonopioid: No Steroid*	Nonopioid: Steroid*	Mean Difference: No Steroid vs Steroid	
Measure	Mean (97.5% CI)	Mean (97.5% CI)	Mean (97.5% CI)	*P* value

				
Satisfaction with pain medication^†,‡^	0.87 (0.80, 0.94)	0.90 (0.83, 0.98)	−0.04 (−0.12, 0.04)	.3
# of analgesic tablets returned^‡^	8.52 (7.10, 9.95)	8.10 (6.52, 9.68)	0.42 (−0.90, 1.74)	.5

Measure	n (%)	n (%)	Odds Ratio (97.5% CI)	*P* value

Emergency clinic visit required^§^	24 (3.9)	9 (3.4)	0.99 (0.95, 1.04)	.7
Postoperative rescue medication prescribed^§^	18 (2.9)	8 (3.0)	1.01 (0.97, 1.05)	.7
Adverse side effect reported in E-diary^§^	539 (86.4)	234 (88.3)	0.98 (0.92, 1.05)	.5
	Opioid Groups			
	Opioid: no Steroid*	Opioid: Steroid*	Mean difference: No steroid vs steroid	
Measure	Mean (97.5% CI)	Mean (97.5% CI)	Mean (97.5% CI)	*P*-value

				
Satisfaction with pain medication^†,‡^	0.79 (0.73, 0.86)	0.86 (0.78, 0.94)	−0.06 (−0.15, 0.02)	.090
# of analgesic tablets returned^‡^	9.89 (8.46, 11.33)	9.42 (7.80, 11.04)	0.47 (−0.88, 1.83)	.4

Measure	n (%)	n (%)	Odds Ratio (97.5% CI)	*P*-value

Emergency clinic visit required^§^	27 (4.3)	7 (2.9)	1.02 (0.97, 1.06)	.4
Postoperative rescue medication prescribed^§^	42 (6.7)	10 (4.1)	1.01 (0.97, 1.06)	.5
Adverse side effect reported in E-diary^§^	580 (91.6)	224 (91.8)	0.99 (0.93, 1.06)	.8

*Note:* Models adjusted for age, sex, race/ethnicity, average pain at preoperative visit, number of teeth extracted, maximum impaction level, method used to extract teeth, type of anesthesia administered, and use of intra-operative antibiotics and intra-operative Marcaine.

* Groups listed in the order they are compared; for example, nonopioid versus opioid is calculated: nonopioid - opioid (mean difference) or nonopioid/opioid (odds ratio).

† Satisfaction with medication was rated using a five-point Likert scale 1 = extremely satisfied, 2 = satisfied, 3 = neither satisfied nor dissatisfied, 4 = dissatisfied, 5 = extremely dissatisfied.

‡ Mixed effect regression model with random effect for site.

§ Logistic regression model with a random effect for site.

Look et al. Intra-OPERATIVE Dexamethasone with Opioids or Non-Opioids? J Oral Maxillofac Surg 2026.

## References

[1] Almeida RD, Lemos CA, Moraes SD, et al. Efficacy of corticosteroids versus placebo in impacted third molar surgery: Systematic review and meta-analysis of randomized controlled trials. Int J Oral Maxillofac Surg 48(1):118–131, 2019 10.1016/j.ijom.2018.05.023 29941229

[2] Markiewicz MR, Brady MF, Ding EL, Corticosteroids reduce post-operative morbidity after third molar surgery: A systematic review and meta-analysis. J Oral Maxillofac Surg 66(9):1881–1894, 2008 10.1016/j.joms.2008.04.022 18718396

[3] Selvido DI, Bhattarai BP, Niyomtham N, Review of dexamethasone administration for management of complications in postoperative third molar surgery. J Korean Assoc Oral Maxillofac Surg 47(5):341–350, 2021 10.5125/jkaoms.2021.47.5.341 PMC8564082 34713808

[4] Parhizkar P, Schmidlin PR, Bornstein MM, Can adjunctive corticosteroid therapy improve patient-centered outcomes following third molar surgery? A systematic review. Med Oral Patol Oral Cir Bucal 27(5):e410–e418, 2022 10.4317/medoral.25177 PMC9445603 35975802

[5] Susarla SM, Blaeser BF, Magalnick D. Third molar surgery and associated complications. Oral Maxillofac Surg Clin North Am 15(2):177–186, 2003 10.1016/S1042-3699(02)00102-4 18088673

[6] American Association of Oral and Maxillofacial Surgeons. Opioid prescribing: Acute and postoperative pain management, 2024. https://aaoms.org/wp-content/uploads/2024/03/opioid_prescribing.pdf. Accessed May 25, 2025

[7] Brattsand R, Linden M. Cytokine modulation by glucocorticoids: Mechanisms and actions in cellular studies. Aliment Pharmacol Ther 10(Suppl 2):81–90, 1996 10.1046/j.1365-2036.1996.22164025.x 8899106

[8] Ngeow WC, Lim D. Do corticosteroids still have a role in the management of third molar surgery? Adv Ther 33(7):1105–1139, 2016 10.1007/s12325-016-0357-y PMC4939150 27287853

[9] Kim K, Brar P, Jakubowski J, The use of corticosteroids and nonsteroidal antiinflammatory medication for the management of pain and inflammation after third molar surgery: A review of the literature. Oral Surg Oral Med Oral Pathol Oral Radiol Endod 107(5):630–640, 2009 10.1016/j.tripleo.2008.11.005 19157919

[10] Sugragan C, Sirintawat N, Kiattavornchareon S, Do corticosteroids reduce postoperative pain following third molar intervention? J Dent Anesth Pain Med 20(5):281–291, 2020 10.17245/jdapm.2020.20.5.281 PMC7644360 33195806

[11] Baxendale BR, Vater M, Lavery KM. Dexamethasone reduces pain and swelling following extraction of third molar teeth. Anaesthesia 48(11):961–964, 1993 10.1111/j.1365-2044.1993.tb07474.x 8250191

[12] De Gea Rico A, Muttoni E, Vassiliou LV, Post-operative pain management in oral and maxillofacial surgery and the formulation of new electronic prescribing order sets. Adv Oral Maxillofac Surg 3:100119, 2021

[13] Van Rensburg R, Reuter H. An overview of analgesics: NSAIDs, paracetamol, and topical analgesics part 1. S Afr Fam Pract 61 (Suppl 1):S13–S18, 2019

[14] Daniels SE, Goulder MA, Aspley S, A randomized, fiveparallel-group, placebo-controlled trial comparing the efficacy and tolerability of analgesic combinations including a novel single-tablet combination of ibuprofen/paracetamol for postoperative dental pain. Pain 152(3):632–642, 2011 10.1016/j.pain.2010.12.012 21257263

[15] Ohashi N, Kohno T. Analgesic effect of acetaminophen: A review of known and novel mechanisms of action. Front Pharmacol 11: 580289, 2020 10.3389/fphar.2020.580289 PMC7734311 33328986

[16] Hyllested M, Jones S, Pedersen JL, Comparative effect of paracetamol, NSAIDs or their combination in postoperative pain management: A qualitative review. Br J Anaesth 88(2): 199–214, 2002 10.1093/bja/88.2.199 11878654

[17] Moore RA, Derry S, Aldington D, Single dose oral analgesics for acute postoperative pain in adults: An overview of cochrane Reviews. Cochrane Database Syst Rev 2015(9):CD008659, 2015 10.1002/14651858.CD008659.pub3 PMC6485441 26414123

[18] Candido KD, Perozo OJ, Knezevic NN. Pharmacology of acetaminophen, nonsteroidal antiinflammatory drugs, and steroid medications: Implications for anesthesia or unique associated risks. Anesthesiol Clin 35(2):e145–e162, 2017 10.1016/j.anclin.2017.01.020 28526157

[19] Feldman CA, Fredericks-Younger J, Desjardins PJ, Nonopioid vs opioid analgesics after impacted third-molar extractions. J Am Dent Assoc 156(2):110–123, 2025 10.1016/j.adaj.2024.10.014 39755971

[20] Huffman GG. Use of methylprednisolone sodium succinate to reduce postoperative edema after removal of impacted third molars. J Oral Surg 35(3):198–199, 1977 264521

[21] Rizqiawan A, Lesmaya YD, Rasyida AZ, Postoperative complications of impacted mandibular third molar extraction related to patient’s age and surgical difficulty level: A cross-sectional retrospective study. Int J Dent 2022:7239339, 2022 10.1155/2022/7239339 PMC8749374 35027927

[22] Vishal, Khaitan T, Ranjan R, Primary closure after surgical extraction of mandibular third molar with or without tube drain: A prospective study. J Fam Med Prim Care 9(2):637–641, 2020 10.4103/jfmpc.jfmpc_1144_19 PMC7114065 32318395

[23] Bello SA, Adeyemo WL, Bamgbose BO, Effect of age, impaction types and operative time on inflammatory tissue reactions following lower third molar surgery. Head Face Med 7:8, 2011 10.1186/1746-160X-7-8 PMC3114767 21527036

[24] Momesso GAC, Lemos CAA, De Lima VN, Is the combination of corticosteroids plus NSAIDs effective for preemptive analgesia after third molars surgery? A systematic review and meta-analysis. Res Soc Dev 9(9):e968997979, 2020

[25] Mercadante SL, Berchovich M, Casuccio A, A prospective randomized study of corticosteroids as adjuvant drugs to opioids in advanced cancer patients. Am J Hosp Palliat Care 24(1):13–19, 2007 10.1177/1049909106295431 17347500

[26] Aminmansour B, Khalili HA, Ahmadi J, Effect of high-dose intravenous dexamethasone on postlumbar discectomy pain. Spine (Phila Pa 1976) 31(21):2415–2417, 2006 10.1097/01.brs.0000238668.49035.19 17023848

[27] Centers for Disease Control and Prevention. U.S. Overdose Deaths Decrease in 2023, First Time Since 2018. National Center for Health Statistics, 2024. https://www.cdc.gov/nchs/pressroom/nchs_press_releases/2024/20240515.htm. Accessed May 25, 2025

[28] Bansal T, Singhal S, Taxak S, Dexamethasone in anesthesia practice: A narrative review. J Anaesthesiol Clin Pharmacol 40 (1):3–8, 2024 10.4103/joacp.joacp_164_22 PMC11042091 38666172

[29] Waldron NH, Jones CA, Gan TJ, Impact of perioperative dexamethasone on postoperative analgesia and side-effects: Systematic review and meta-analysis. Br J Anaesth 110(2):191–200, 2013 10.1093/bja/aes431 PMC3544008 23220857

[30] Majid OW, Mahmood WK. Use of dexamethasone to minimise post-operative sequelae after third molar surgery: Comparison of five different routes of administration. Oral Surg 6 (4):200–208, 2013

